# Localised residency and inter-annual fidelity to coastal foraging areas may place sea bass at risk to local depletion

**DOI:** 10.1038/srep45841

**Published:** 2017-04-04

**Authors:** Thomas K. Doyle, Damien Haberlin, Jim Clohessy, Ashley Bennison, Mark Jessopp

**Affiliations:** 1 Zoology, School of Natural Sciences, Ryan Institute, National University of Ireland Galway, Ireland; 2 MaREI Centre, National University of Ireland Galway, Ireland; 3 MaREI Centre, Environmental Research Institute, University College Cork, Ireland; 4 School of Biological, Earth and Environmental Sciences, University College Cork, Ireland; 5 Cork Harbour Angling Hub, Cobh, Cork, Ireland

## Abstract

For many marine migratory fish, comparatively little is known about the movement of individuals rather than the population. Yet, such individual-based movement data is vitally important to understand variability in migratory strategies and fidelity to foraging locations. A case in point is the economically important European sea bass (*Dicentrarchus labrax* L.) that inhabits coastal waters during the summer months before migrating offshore to spawn and overwinter. Beyond this broad generalisation we have very limited information on the movements of individuals at coastal foraging grounds. We used acoustic telemetry to track the summer movements and seasonal migrations of individual sea bass in a large tidally and estuarine influenced coastal environment. We found that the vast majority of tagged sea bass displayed long-term residency (mean, 167 days) and inter-annual fidelity (93% return rate) to specific areas. We describe individual fish home ranges of 3 km or less, and while fish clearly had core resident areas, there was movement of fish between closely located receivers. The combination of inter-annual fidelity to localised foraging areas makes sea bass very susceptible to local depletion; however, the designation of protected areas for sea bass may go a long way to ensuring the sustainability of this species.

Fisheries investigations have revealed the general migratory patterns for many commercial marine fish, with broadcast spawners typically alternating between spawning, foraging and overwintering areas^1^. For many species our knowledge derives from the exploration of extensive fisheries data (landing of adults and juveniles) and egg surveys. These data provide snapshots of the populations’ migratory movements as a whole, rather than detailed information on individual behaviour^2^,^3^. However, such data may also be biased towards fisheries effort rather than fish movements *per se*^4^. While this broadscale population information is vital for stock assessments and fisheries management, defining the movements of individuals rather than the population as a whole can provide important information on variability in fish behaviour and migratory strategies. Indeed, research using telemetry and otolith microanalysis has shown that the migratory patterns and associated life cycles for marine fish can be “complex adaptive systems” with some individuals migrating and others being entirely resident^5^,^6^,^7^,^8^.

In the Northeast Atlantic, European sea bass (*Dicentrarchus labrax*) inhabit the coastal waters of Ireland, England, Wales, France and the Netherlands during the summer months^9^,^10^,^11^. They are a long-lived fish (>20 years)^12^ and are known to feed on a variety of prey items including shore crabs (*Carcinus*), shrimp (*Crangon*), juvenile plaice and flounder^12^. Sea bass are also known to shoal at the surface in pursuit of bait fishes (*Clupea harengus* or *Sprattus sprattus*)^13^. During autumn and winter, sea bass migrate out of their coastal habitats to spawn and overwinter before returning to coastal foraging locations in spring or early summer. Evidence from previous mark-recapture studies found that many sea bass return to the same areas in successive years, with one study showing that 55% of all recaptured fish were within 16 km of their original tagging location^9^. Despite this knowledge, we still have little information on the composition of local populations at coastal foraging grounds, including whether they are transient or resident at specific sites for extended periods, the scale of local movements and level of intra and inter-annual site fidelity, and whether the timing of migration is driven by intrinsic or extrinsic factors.

Sea bass are an economically important species with the combined commercial and recreational fishing mortality well above the maximum sustainable yield^14^. While the species is not managed under the Common Fisheries Policy, in early 2015 and again in 2016, the EU Commission introduced a series of emergency measures to halt population declines, including catch restrictions and an increase in the minimum landing size from 36 to 42 cm. The EU Commission has since proposed that further measures will be adopted to protect the species. However, without detailed information on the susceptibility of the species to fisheries impacts, the effectiveness of such conservation measures may not be maximised.

Here we used acoustic telemetry to track the summer movements and seasonal migrations of individual sea bass. Specifically, we aimed to test the hypotheses that during summer: (1) sea bass are locally resident in coastal areas, (2) sea bass exhibit limited spatial movement in coastal areas, and (3) that individual fish have high site-fidelity, returning to the same foraging areas in successive years.

## Results

**Figure 1:**
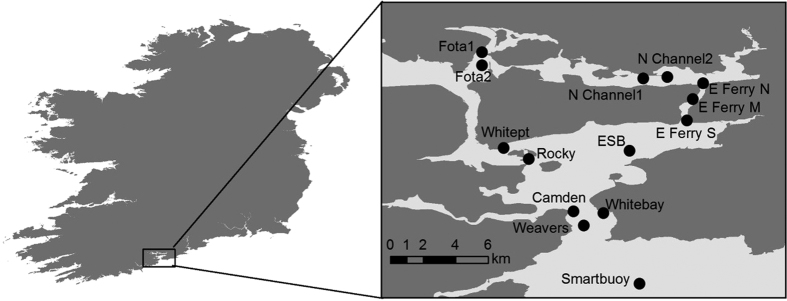
Map of study area showing receiver locations. Map was generated in ArcGIS 10.2 (http://www.esri.com/software/arcgis/arcgis-for-desktop).

**Figure 2:**
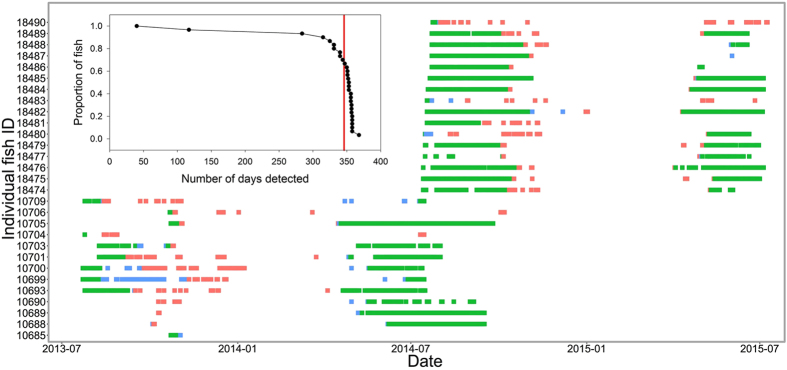
Temporal occurrence of sea bass (fish tagged in summer 2013 bottom left, summer 2014 top right) in Cork harbour. Each line represents the daily detections of one fish. Green indicates that the fish was detected by the inner harbour receivers; blue indicates transitional receivers between inner and outer harbour and red indicates the outer harbour receivers. Inset shows survival of tagged fish over time, noting that 90% of fish were still detected by the acoustic array after 315 days (very close to expected tag battery life–vertical red line).

Sea bass tagged and released in Cork harbour (Fig. 1) (n = 30, mean total length 54.4 ± SD 9.3 cm, mean weight 1910 ± 1071 g) had a high apparent survival rate post-tagging, with 100% of fish still alive after 30 days (Table 1). Ninety-seven percent of fish were detected after 90 days, and 90% of fish were still detected by the acoustic array after 315 days (Fig. 2). The four shortest tracking periods were 40, 117, 284, and 315 days at large.

A total of 467,814 acoustic tag detections were recorded across the study period. There was considerable variation in the number of detections per fish (range 601 to 62,934, mean 15,594 ± 14,924). The vast majority of detections were near the release sites (East and West harbour, 451,372 or 97%) with only 16,486 detections (3%) at the outer boundary of the harbour array. Far more detections were received in the East (381,009; 82%) where more fish were tagged than the West (68,222, 18%) harbour tagging areas (20 vs. 10) and had wider receiver coverage (5 vs. 2).

### Harbour Residency and migration

The mean Harbour Residency (HR, see methods for definition of HR) over the tagging period was 167 ± 57 days (Table 1). The maximum HR by any fish was 275 days (fish #10706) but six fish were resident for 200 days or more. Twenty-seven fish were resident for 100 days or greater (Table 1). The mean HR increases to 173 ± 53 days when the two fish that did not return to the harbour after migration are removed.

In 2013, there was a protracted period of 15 weeks during which fish left the Cork Harbour acoustic array. The majority left during weeks 40–45 (October to November) while two fish were still intermittingly resident until week 2 the following year (see Fig. 2 for indicative departure and arrival times of individual fish). In 2014, all fish bar one left the harbour within a six week period (weeks 41–46), with the remaining fish leaving Cork harbour in week 1 of 2015. All fish departing the harbour in 2013 returned to the harbour within a 12 week period in 2014 (weeks 12–23), with 1 or 2 fish each week. All fish tagged in 2014 returned to the harbour within a 10 week period in 2015 (weeks 14–23), although most of these returned during a six week window (weeks 14–19). The mean date for fish leaving and returning to the harbour was mid-October and mid-April, respectively.

### Single Receiver Residency and fish movements within the harbour

**Figure 3:**
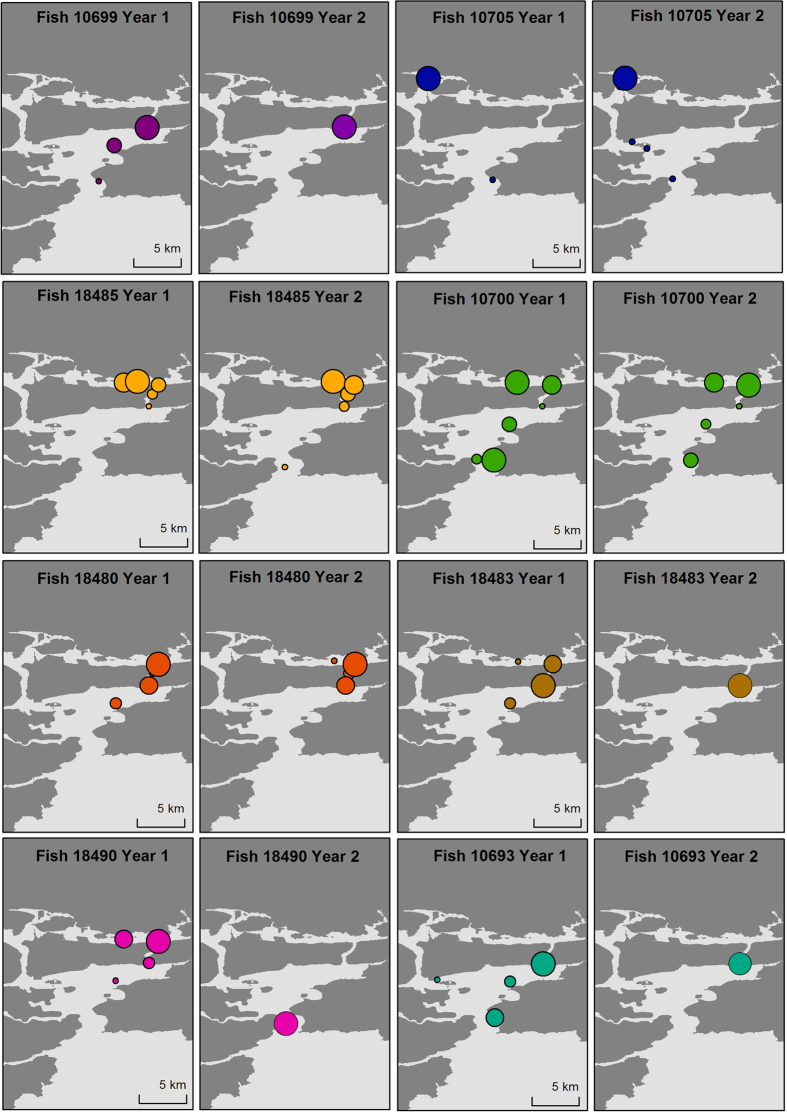
Spatial occurrence of sea bass within Cork Harbour, Ireland. Fish ID corresponds to Tables 1 and 2. Coloured bubbles indicate the relative total residency at acoustic receivers within years. Left panels show fish residency after tagging, and right panels show residency after winter migration. Arrows indicate general movements between receivers. Star indicates capture location. Map was generated in ArcGIS 10.2 (http://www.esri.com/software/arcgis/arcgis-for-desktop).

Four thousand two hundred and fifty-nine individual Single Receiver Residency (SRR, see method for definition) events were detected (mean 142 ± 102 per fish). Forty-four percent of all residency events ended when a fish was detected at another receiver, with the remainder attributed to timeout events where the fish was not detected by any receiver for six hours. Individuals were resident across receiver locations for a mean total of 1349 (±943) hours, but most fish spent a large proportion of their SRR at one particular receiver. The mean proportion of total SRR time at the most visited receiver was 0.80 ± 0.20 (Table 2). For example, fish #10701 spent 86% of its SRR time at East Ferry North receiver, and fish #10705 spent 99% of its SRR time at Fota 2 receiver (Fig. 3). The mean duration of SRR events varied between individuals (range 2–39 hours), with fish resident on average 12 (±9) hours per residency event. Fish #10688 only had 70 SRR events, each event lasting on average 27 hours, and a maximum SRR event of 188 hours during its 118 days present in the harbour. The longest SRR event lasted 1626 hours (68 days) by fish #18485.

The average duration of residency also varied considerably across stations; fish spent comparatively little time at Camden (mean 1.9 hours, n = 6) and ESB (mean 4.1 hours, n = 12), longer at Whitebay (mean 4.6 hours, n = 28) and Fota 2 (mean 7.8 hours, n = 10), but considerably longer at East Ferry South (mean 10.4 hours, n = 23) and East Ferry North (mean 19.6 hours, n = 16). Interestingly, while average residency at East Ferry South was only 10.4 hours, this receiver was the most visited receiver in the network, with residency events from 23 fish being detected, suggesting this is an important area, or a key transiting point in the harbour.

**Figure 4:**
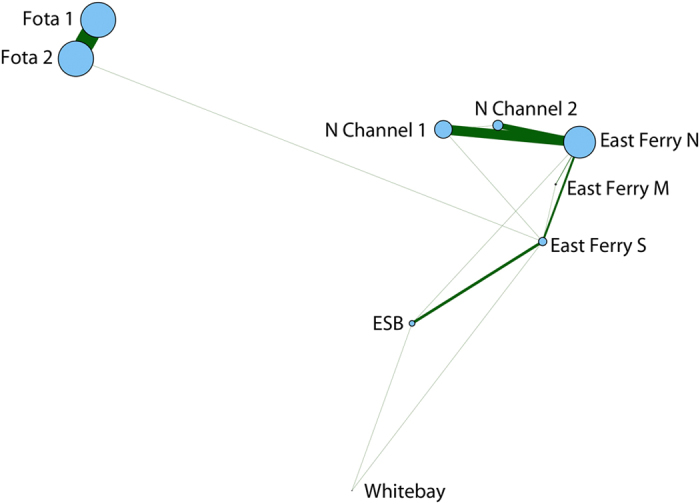
Spatial Network map showing movements of tagged fish between receivers during summer months (June–August). Receiver locations represent distances between adjacent receivers and are scaled by the number of repeat residency events at that receiver (i.e. separate residency events with no residency detected at another receiver in the intervening period). Lines connecting receivers are scaled by the number of movements between receivers.

Most fish tended to be very locally resident, spending most of their time at or near one or two receivers in the inner harbour (Fig. 3). This was shown in the network analysis, with large numbers of repeat residency events occurring at key receivers within the network, notably, Fota 1 and 2, and East Ferry North, each with over 100 repeat residencies (Fig. 4). However, fish did exhibit movements between receivers during the summer harbour residency. Connections between closely located receivers accounted for most movements between receivers within the array (Fig. 4). In particular, there were 226 movements of fish between the closely sited Fota 1 and Fota 2 receivers, and 217 movements between East Ferry North and its adjacent receivers. While few repeat residency events occurred at East Ferry South, this was the most connected receiver, with movements between it and 6 other receivers in the network during summer. Of note is a single movement between East Ferry South and Fota that bypassed intermediate receivers at ESB, Whitepoint, and Rocky Island. This was fish #10688 that moved from East Ferry South to Fota on 6^th^ June 2014, and was likely associated with return migration, as this fish was first detected back in the network following winter migration on 4^th^ June 2014. Other movements may be attributed to more mobile fish such as fish #10709, which moved between Whitebay, ESB and East Ferry South multiple times between late August and October, and spent the majority of its summer distribution in this general area with the exception of 2 detections at East Ferry North.

### Inter-annual site fidelity

Twenty-eight fish out of thirty (93%) returned to Cork harbour following migration. Of these 28 fish, 24 (86%) returned to the same area (East or West) that they had occupied prior to migration, highlighting high inter-annual site fidelity (Fig. 3). Twenty (77%) returned to the exact receiver they were previously most frequently recorded at, and continued to use this site as their primary residence. Fish #18490 was the most transient/mobile fish, being resident for short periods at a large number of receivers, and changing the area of highest use from the more northerly receivers to the harbour entrance in year 2 (Fig. 3).

## Discussion

Using two different measures of residency, our results show that 24 out of 30 acoustically-tagged sea bass displayed long term residency in Cork harbour and that within this area fish displayed fine-scale residency and fidelity to specific sites. For example, 80% of sea bass spent >60% of their total residency time (SRR) at one particular receiver (typically the receiver nearest to where the fish was originally caught). If the residency time spent at the next nearest receiver is included, 80% of sea bass spent >90% of their total residency time between these two receivers (located max 3 km apart). While such fine-scale residency is better described for tropical reef fish that maintain a territory for breeding or cultivation (e.g. grazing reef fish^15^), for marine migratory fish (teleosts), comparatively little is known about the size of their foraging area or how much time they spend at particular foraging locations^16^. This dearth in knowledge is largely due to the difficulty in making frequent repeated observations of individual fish at sea where the logistics and costs of such endeavours can be prohibitive^3^. Some exceptions include a recent study that used acoustic telemetry to show that cod (*Gadus morhua*) were highly resident within an offshore wind farm in the North Sea^17^, and that yellowfin bream (*Acanthopagrus australis*) displayed a surprising degree of fidelity to very localised areas (0.1 km^2^) over four 3-day tracking periods^18^. Like sea bass, yellowfin bream adults are thought to leave estuarine environments in winter to move to coastal zones such as surf zones for spawning.

We found that there was extremely limited movement of individual fish between the East and West harbour areas. The only exceptions were two West harbour fish that were detected by one or more East harbour receivers after returning from their migration, and two East harbour fish that were briefly detected by either White Point or Rocky receivers. No East harbour fish moved up along the Western Channel to either Fota receivers. Such limited harbour movements between tagging areas, suggests that most sea bass are residents with small home ranges of less than 3 linear km. Like many other fish, within this home range sea bass may have core areas that are used for resting or foraging on a daily basis^19^. However, very few fish displayed what could be described as transient behaviour. Two possible exceptions include fish #18490 that left Cork harbour 7 days after being tagged and when it returned the following year after the winter migration, was only detected by receivers at the entrance to the harbour (Fig. 3). The second transient fish (fish #18483) was never resident at any receiver and was not detected for large periods post tagging nor when it returned briefly to the harbour post-migration. These fish were at the smaller end of the size distribution of tagged fish (both <46 cm long), consistent with the findings of Pickett *et al*.^11^ who described how sea bass disperse chiefly during their adolescent phase.

Most sea bass spent six months (mean 167 ± 57 days) in the harbour. Such long term residency supports the theory that estuarine and coastal areas may play a critical role in the life cycle of long-lived fish, as this is where most growth will occur^20^. Cork harbour is a highly productive ecosystem with large areas of intertidal mudflats fringed by salt marshes and rocky areas with brown algae. In France, such salt marsh areas are known to play a fundamental role in the feeding of 0-group sea bass, where juveniles can consume on average 8% of their body weight during each tide^21^. In this study, adults were resident in several different habitats: (1) in shallow waters through a dense canopy of brown algae (*Fucus* sp.), (2) over an area of large mussel beds with fast flowing water, and (3) in a comparatively deep body of water (5–10 m) with fast flow rates. Such different foraging areas suggest that sea bass are highly adaptable and have different feeding strategies for exploiting different environments, possibly in response to changing feeding conditions with tide.

While inter-annual fidelity to foraging locations is known in many large marine vertebrates^22^,^23^,^24^, relatively few studies have documented inter-annual fidelity to foraging locations in migratory marine fish. For sea bass such behaviour was previously documented at a spatial scale of ~16 km with some individual fish being recaptured at the same tagging/release site many years later. For example, Pawson *et al*.^9^ described 17 fish being recaptured at their original tagging locations in south Wales and southwest England during the period 2000–2006. However, it is not certain how prevalent this homing behaviour is within the population. Here we conclusively show that 93% of tagged sea bass that returned to Cork harbour, did so to very localised coastal foraging areas (i.e. East or West harbour areas). Furthermore, of these twenty-six fish, 77% (20) returned to and were resident at the exact receiver that they were most resident at before migrating; demonstrating the fine spatial scale of inter-annual site fidelity. Similar inter-annual fidelity to foraging grounds has been documented for few other marine teleosts^25^,^26^. It is interesting to note that the closely related striped bass (*Morone saxatilis* W) of North America has taken site fidelity to the extreme, with some adults remaining resident in estuarine areas year round^8^. While Pawson *et al*.^27^ found that some adolescent sea bass in the Thames estuary were resident all year round, we found no evidence of partial migration, but note that the majority of tagged fish in our study were larger than the adolescent fish tracked in the Thames estuary.

An interesting observation from this study was that one fish returning to its resident foraging area (Fota 2), took a long detour up the East harbour area (18 days) before navigating its way back to Fota 2 (fish #10690). While this is only one example of an aberrant return migration, it suggests that sea bass have a strong sense of home and must be using several cues to navigate around the harbour that may sometimes go wrong. The fact that East Ferry South receiver had very few ‘repeat’ residencies but was the most connected ‘node’ in the network of receivers (Fig. 4), suggests that this location was used as a transiting point and may point to either topological cues based on bathymetry, or chemical cues based on the source of freshwater input to the harbour.

Our study clearly demonstrates that the vast majority of tagged sea bass displayed long-term residency and inter-annual fidelity to specific sites. Assuming this tendency of residency is a general behaviour for sea bass across their range, our findings have important implications for the species’ susceptibility to local depletion. In addition to the combined commercial and recreational fishing mortality being well above the maximum sustainable yield^14^, illegal, unreported, and unregulated (IUU) fishing for sea bass is an issue in Ireland and other EU countries. Therefore, it is important that EU emergency measures to halt the decline in sea bass are widened to consider the effect of removing highly localised fishes. It is also important for fisheries managers and policy makers to stress the importance of measures such as the ‘catch and release’ limit of 1 fish per day, which effectively minimises the impact of recreational fishing. Given the localised residency of sea bass, the designation of protected areas for sea bass proposed by Pawson *et al*.^9^ and Cambie *et al*.^28^ may be a particularly effective means of conservation to ensure sustainability of this species.

## Methods

### Study site

Sea bass were tracked within Cork harbour, a large sheltered bay system that encompasses several river estuaries (most notably the River Lee), large islands, channels and inlets, located on the southwest coast of Ireland (Fig. 1). Cork harbour has a large tidal range (4.5 m) and has a convoluted coastline with an intertidal area of 1,460 ha (14.6 km^2^) dominated by mudflats fringed by salt marshes (cordgrass) and gentle sloping shores. Capture and tagging of sea bass was carried out in the east and west of the harbour. Specific capture locations within each area were selected on the basis of previous successful sea bass angling. In the East harbour, capture was focussed in the East Channel and North Channel, while in the west, capture was at Fota. The North Channel is a 9 km long, shallow (1–10 m) channel draining into the East Channel, but with a small connection to the Fota area on the west of the harbour that is disconnected during extreme low tides. The East Channel is a, 5–15 m deep fast flowing body of water during ebb and flood periods of the tide. The Fota location is a very shallow body of water with extensive mudflats and smaller areas of mussel beds. A large section of the Fota area is entirely exposed during low tides.

### Acoustic tracking

In June 2013, 10 Vemco VR2W acoustic receivers were deployed in capture locations and at entrances to channels to maximise chances of detecting fish. An additional three receivers were added to the array in July 2014 (after some were lost to extreme storm activity during winter 2013), and a Vemco VR2C (cabled) receiver was deployed outside Cork harbour in January 2014. A range test confirmed that 88.6% of acoustic transmissions were detected by a receiver located 300 m from the transmitter.

During the summers of 2013 and 2014, 34 sea bass were captured by rod and line using soft plastic lures, flies and plugs by local anglers. Two fish were released without tagging because they were undersized (<40 cm) and two fish died during the tagging procedure (did not recover from the anaesthetic). The remaining 30 fish were captured at 4 locations: East Ferry South (n = 5 in 2013, n = 1 in 2014), East Ferry North (n = 3 in 2013, n = 6 in 2014), Fota 2 (n = 6 in 2013, n = 4 in 2014), and North Channel 2 (n = 5 in 2014) (Fig. 1 and Table 1). Once captured, each fish was placed in a 40-litre bucket of sea water and transferred to a land-based processing station. Each fish was anaesthetised with phenoxyethanol (0.5 ml per litre of sea water) in a large tank until they lost equilibrium and did not respond to a tail pinch^29^. Fish were then placed upside-down in a V-shaped cradle with seawater pumped into the mouth to ensure a good supply of oxygenated water over the gills. A VEMCO acoustic tag (V9, 69 KHz, random delay 60–180 seconds, expected life expectancy 347 days) was surgically implanted into the body cavity of each fish by making a small incision (10–15 mm long) posterior to the pelvic fins and anterior to the anus. The opening was closed using synthetic absorbable sutures (polyamide monofilament, DS19 3/0, Dafilon). An external T-bar Floy tag was also attached just below the dorsal fin so that recaptured fish could be identified by local anglers and returned to sea. Each fish was placed in a recovery tank for 5–15 mins before being released at the initial capture location. It was not possible to reliably determine the sex of the fish during the tagging procedure.

No aquarium trials were carried out prior to tagging to investigate impact of tags as previous studies have demonstrated that internally implanted tags did not show any effect when compared to untagged controls^29^. All animal tagging procedures were approved by the Animal Welfare Body (AWB) and the Animal Experimental Ethics Committee (AEEC) of University College Cork. All sea bass were tagged under license (AE191130/I007, AE19130/P001) issued by the Irish Health Products Regulatory Authority (HPRA) and complied with the EU Directive 2010/63/EU for scientific research on animals.

### Definition of residency of site fidelity

For the purposes of this study, residency is defined as an “individual exhibiting largely uninterrupted occupancy of a limited area for a specified period of time”^30^. Site fidelity is defined as “the return of an individual to a location where it previously resided after having left it for some defined period (months) of time”^30^.

To assess the degree and scale of residency, we used two different measures of residency based on scale. (1) Harbour Residency (HR) is equal to the total number of days a fish was present within the entire acoustic array of Cork harbour. HR began when a fish was tagged, was broken when a fish left the harbour, re-continued once a fish returned to the harbour and ended when detections of that fish ceased (typically close to anticipated transmitter battery life) or if the fish left the harbour again. During such residency, a fish may be present in the harbour but not detected by the array i.e. a fish could be between receivers. (2) Single Receiver Residency (SRR) is equal to the duration of time in hours a fish was detected at a single receiver (~0.8 km^2^). A fish was considered to begin a period of SRR residency after 2 detections were made at a receiver, and ended either when a fish was detected at another receiver, or when no signal had been received for a period of 6 hours, to account for any effects of tide on probability of detecting acoustic signals^31^. Acoustic detections were compiled and analysed in the R statistical framework using the package VTrack^32^.

Because no fish were reported as dead or recaptured by anglers or fishermen, survival rates of sea bass were estimated based on detections within the acoustic array. While we were unable to differentiate between tag failure and mortality, any fish that ceased being detected within 90% of the expected tag life were assumed dead. To examine and visualise the main movements by fish within the array, we conducted a network analysis using the *igraph*^33^ package in R. Detections from summer months June-August were used to exclude movements associated with migration in and out of the harbour and construct a spatial graph of movements within the harbour with ‘connections’ weighted by the number of movements between receivers.

### Inference of migration

Increased receiver coverage at the harbour entrance maximised detection of fish leaving and entering the harbour. Outward migration is illustrated by detections occurring in highly resident areas of the inner harbour throughout the summer, and transitioning to detections towards the harbour entrance during autumn/winter prior to an absence of detections over winter. The reverse pattern of detections at the harbour entrance transitioning to highly resident areas of the inner harbour in spring illustrates return migration (see Fig. 2).

### Use of experimental animals and human subjects

Sea bass were tagged under license (AE191130/I007, AE19130/P001) issued by the Irish Health Products Regulatory Authority (HPRA) and complied with the EU Directive 2010/63/EU for scientific research on animals. All animal tagging procedures were approved by the Animal Welfare Body (AWB) and the Animal Experimental Ethics Committee (AEEC) of University College Cork.

## Additional Information

**How to cite this article:** Doyle, T. K. *et al*. Localised residency and inter-annual fidelity to coastal foraging areas may place sea bass at risk to local depletion. *Sci. Rep.* **7**, 45841; doi: 10.1038/srep45841 (2017).

**Publisher's note:** Springer Nature remains neutral with regard to jurisdictional claims in published maps and institutional affiliations.

## Figures and Tables

**Table 1:** Summary statistics of acoustic monitoring data for 30 tagged sea bass.

Fish ID	Tagging location	Total length (cm)	Date tagged	Date fish left for seasonal migration	Date fish returned after migration	Date last detected	Total number of detections	No of days detected by the array	Days at liberty	Harbour residency
10685	FOTA 2	63.0	24/09/2013	02/11/2013	No return	02/11/2013	755	8	40	40
10686	FOTA 2	62.0	24/09/2013	22/10/2013	03/06/2014	26/09/2014	4444	105	368	145
10688	FOTA 2	61.0	24/09/2013	06/10/2013	04/06/2014	16/09/2014	9048	108	358	118
10689	FOTA 2	62.5	24/09/2013	11/10/2013	07/05/2014	16/09/2014	3581	126	358	151
10690	FOTA 2	51.0	24/09/2013	01/11/2013	30/04/2014	05/09/2014	7810	59	347	168
10693	EFERRYS	47.5	25/07/2013	12/12/2013	05/04/2014	16/07/2014	24240	156	357	244
10699	EFERRYS	40.5	23/07/2013	20/12/2013	04/06/2014	15/07/2014	17441	163	358	193
10700	EFERRYN	41.0	23/07/2013	08/01/2014	30/04/2014	13/07/2014	11410	125	356	245
10701	EFERRYN	50.0	09/08/2013	18/12/2013	24/03/2014	01/08/2014	15122	125	358	263
10703	EFERRYS	62.0	09/08/2013	26/10/2013	07/05/2014	01/08/2014	10966	121	358	166
10704	EFERRYS	53.0	25/07/2013	28/08/2013	11/07/2014	15/07/2014	601	14	356	40
10705	FOTA 2	63.0	04/10/2013	04/11/2013	16/04/2014	25/09/2014	13623	175	357	195
10706	EFERRYN	45.0	22/10/2013	02/01/2014	20/03/2014	07/10/2014	4367	135	351	275
10709	EFERRYS	55.4	25/07/2013	03/11/2013	23/04/2014	15/07/2014	17433	115	356	186
18474	EFERRYN	50.0	14/07/2014	11/11/2014	10/05/2015	03/06/2015	13882	98	325	146
18475	EFERRYN	47.5	14/07/2014	05/11/2014	14/04/2015	01/07/2015	33817	165	353	194
18476	EFERRYS	78.0	14/07/2014	05/11/2014	03/04/2015	05/07/2015	9590	150	357	209
18477	EFERRYN	65.0	16/07/2014	06/10/2014	29/04/2015	20/06/2015	4886	46	340	136
18479	EFERRYN	62.5	16/07/2014	07/10/2014	12/04/2015	30/06/2015	43192	119	350	164
18480	EFERRYN	48.0	16/07/2014	13/11/2014	08/05/2015	20/06/2015	36791	144	340	165
18481	N CHA 2	50.0	18/07/2014	11/11/2014	No return	11/11/2014	12979	85	117	117
18482	N CHA 2	49.5	18/07/2014	02/01/2015	11/04/2015	04/07/2015	31296	207	352	254
18483	N CHA 2	44.5	18/07/2014	19/11/2014	02/05/2015	26/06/2015	984	17	344	181
18484	N CHA 2	54.0	18/07/2014	13/10/2014	20/04/2015	05/07/2015	38633	159	353	165
18485	N CHA 2	49.0	18/07/2014	04/11/2014	25/04/2015	05/07/2015	62934	167	353	182
18486	FOTA 2	73.5	23/07/2014	14/10/2014	29/04/2015	02/05/2015	1836	79	284	88
18487	FOTA 2	63.0	23/07/2014	04/11/2014	02/06/2015	02/06/2015	9521	105	315	106
18488	FOTA 2	51.0	23/07/2014	20/11/2014	01/06/2015	18/06/2015	7590	84	331	139
18489	FOTA 2	46.5	23/07/2014	10/11/2014	02/05/2015	18/06/2015	16298	124	331	159
18490	EFERRYN	45.0	24/07/2014	31/10/2014	06/05/2015	09/07/2015	2744	35	351	165
	**Mean**	**54.5**					**15594**	**111**	**329**	**167**
	**SD**	9.4					14924	51	71	57

**Table 2:** Summary statistics of Single Receiver Residency data for 30 tagged sea bass.

Fish ID	Tagging location	Total duration of SRR (hours)	No. of SRR events	Mean duration of SRR events (hours)	Max duration of SRR events (hours)	No. of receivers detected at	Proportion of total SRR time at most visited receiver	Most visited receiver	Mean time between SRR receivers (hours)
10685	FOTA 2	106	5	21	56	2	1.00	FOTA2	N/A
10686	FOTA 2	879	132	7	67	3	0.99	FOTA2	0.22
10688	FOTA 2	1865	70	27	188	6	0.97	FOTA2	16.53
10689	FOTA 2	1283	186	7	67	5	0.99	FOTA2	20.40
10690	FOTA 2	706	96	7	111	9	0.50	FOTA2	12.14
10693	EFERRYS	1758	145	12	107	5	0.99	EFERRYS	14.99
10699	EFERRYS	2093	171	12	155	4	0.75	EFERRYS	10.63
10700	EFERRYN	814	162	5	104	6	0.51	EFERRYN	15.80
10701	EFERRYN	1258	128	10	118	6	0.86	EFERRYN	105.09
10703	EFERRYS	1488	129	12	103	3	0.99	EFERRYS	5.81
10704	EFERRYS	54	27	2	13	3	0.57	CAMDEN	49.45
10705	FOTA 2	255	176	14	255	6	0.99	FOTA2	4.31
10706	EFERRYN	2378	156	4	63	8	0.61	N CHA 1	61.18
10709	EFERRYS	1411	112	13	216	6	0.97	EFERRYS	16.11
18474	EFERRYN	1126	186	6	129	6	0.64	EFERRYN	5.45
18475	EFERRYN	3321	85	39	478	4	0.98	EFERRYN	1.48
18476	EFERRYS	1097	184	6	82	4	0.99	EFERRYS	4.61
18477	EFERRYN	398	61	7	142	8	0.64	EFERRYN	49.69
18479	EFERRYN	2222	135	16	212	6	0.92	EFERRYN	2.00
18480	EFERRYN	2787	113	25	703	6	0.97	EFERRYN	5.14
18481	N CHA 2	1296	172	8	45	6	0.38	N CHA 1	1.39
18482	N CHA 2	2558	366	7	140	8	0.55	EFERRYN	7.57
18483	N CHA 2	69	22	3	14	6	0.69	EFERRYS	221.62
18484	N CHA 2	2640	103	26	963	6	0.80	N CHA 2	1.28
18485	N CHA 2	3023	100	30	1626	7	0.83	N CHA 2	1.89
18486	FOTA 2	337	109	3	17	5	0.96	FOTA2	7.60
18487	FOTA 2	1017	148	7	65	4	0.99	FOTA2	0.35
18488	FOTA 2	552	205	3	69	4	0.53	FOTA1	0.55
18489	FOTA 2	1479	541	3	32	4	0.73	FOTA1	0.67
18490	EFERRYN	204	34	6	48	6	0.75	EFERRYN	0.99
	**Mean**	**1349**	**142**	**12**	**213**	**5.4**	**0.80**		**22.24**
